# Rapid source forgetting across modalities: A problem for working memory models

**DOI:** 10.3758/s13421-024-01664-y

**Published:** 2024-12-02

**Authors:** Molly A. Delooze, Dominic Guitard, Nelson Cowan, Candice C. Morey

**Affiliations:** 1 Department of Psychology, Cardiff University, 70 Park Place, Cardiff, CF10 3AT UK; 2 Psychological Sciences, University of Missouri, Columbia, MO USA

**Keywords:** Forgetting, Short-term memory, Working memory, Verbal, Visual

## Abstract

Working memory is a cognitive system that enables the temporary retention (usually a few seconds) of a limited amount of information. However, recent evidence has posed challenges to the conventional understanding of working memory's persistence. Chen et al. (*Psychological Science, 29*(4), 645–655, 2018) demonstrated that participants can easily make judgments using a stimulus’s identity but cannot recall from which source the information came (presented either as a written word or a color patch) just milliseconds earlier. This “Source Amnesia” carries substantial implications for working memory models but has yet to be explored within the realm of verbal information. We fill this gap by investigating the robustness and generalizability of this rapid forgetting phenomenon. We first replicate the observed effect within the visual domain (Experiment 1) and subsequently extend it to the verbal domain (Experiment 2). Finally, we test the idea that participants may instead encode a positional context (Experiment 3), in line with the Interference model (Oberauer & Lin, *Psychological Review, 124*(1), 21, 2017). Aligning with the work of Chen et al. (*Psychological Science, 29*(4), 645–655, 2018), our results consistently reveal a pronounced tendency for rapid forgetting, for both visual and verbal information regardless of whether the information is elicited for recall by format or position cues. The theoretical implications of these findings for current memory models are discussed.

## Introduction

Working memory is the cognitive system which allows us to store and process a limited amount of information necessary to carry out a wide variety of complex acts (Cowan, 2017). Given the capacity limitation of working memory, which is assumed by most models, forgetting, at least temporarily, is vital. For instance, without forgetting, the mind would quickly become overwhelmed and unable to focus on the information most relevant for our current goal. Some pieces of information must be discarded. Therefore, a large part of understanding memory is understanding the circumstances under which we do not remember: forgetting.

Various models have differing approaches to explaining the flow of information into and out of working memory, including how and when forgetting occurs. The Multicomponent model of working memory (originally presented in Baddeley & Hitch, 1974, but see Baddeley, Hitch, & Allen, 2021, for an updated overview) suggests that information is lost from modality-relevant temporary storage systems when we try to exceed their limited storage capacity. The Time-Based Resource-Sharing (TBRS) model (originally presented in Barouillet, Bernardin, & Camos, 2004, but see Barouillet & Camos, 2021, for an updated overview) is one of many models historically which outline that forgetting occurs as a result of time-based decay, wherein the probability to recall an item is reduced as a function of time passing (for another example of a decay and rehearsal account, see also Baddeley et al.’s (1975) account of the Phonological Loop). In the TBRS specifically, this decay occurs only when attention is directed away from the target item. Somewhat similarly, the Embedded Processes model (originally presented in Cowan, 1988, but see Cowan, Morey, & Naveh-Benjamin, 2021, for an updated overview) also states that items may be lost from passive short-term storage through time-based decay, or alternatively by interference from a similar subsequently encoded item. In their Interference model of working memory (Oberauer & Lin, 2017, 2023; see also Oberauer, 2021), Oberauer and Lin consider forgetting to be solely a result of interference: this occurs when the target memory representation is not selected for recall due to competing activation of non-target representations with similar or overlapping context retrieval cues. This theory of forgetting therefore relies on the target having a similar context to the non-targets which are recalled in its place. Popov and Reder’s (2020) Resource-Depletion theory of working memory states that we have a limited pool from which to draw resources for cognitive processing and memory encoding. Each processing or encoding action depletes this pool until insufficient resources are available to encode items so that they can be recalled later. Therefore, Popov and Reder (2020) propose that limits in working memory arise at encoding: once the encoding resource has been depleted by encoding some information, further information cannot be encoded until the resource has had time to recover.

Forgetting is especially fascinating for cases wherein, intuitively, we would firmly expect to remember. Discrepancies exist in estimates for the maximum duration of working memory persistence, with some sources suggesting that items can endure up to 30 s before being transferred to long-term storage (Atkinson & Shiffrin, 1971), others suggesting that the vast majority of items are lost by 18 s (Peterson & Peterson, 1959), and still others suggesting that the life of a working memory representation could be as short as 4 s (Sligte et al., 2008). Despite these differences, it is safe to say that most researchers would not expect attended information to be lost within 1 s. These entrenched expectations mean recent findings concerning the phenomena of rapid forgetting known as “Attribute Amnesia” are particularly problematic for working memory models. Chen and Wyble (2015, 2016) demonstrated attribute amnesia, the apparent forgetting of features less than 1 s after they had certainly been attended. In their paradigms, participants very briefly saw an array of colored characters, with the task to find the letter among the numbers and were only asked to report the target’s location. After many such trials, Chen and Wyble surprised participants by asking them about the identity or the color of the target, and participants responded poorly on these surprise tests. This finding is particularly surprising because the participants must have attended to the identity of the target to be able to identify it as the letter among numbers, yet they seem to be very quickly unable to recall which letter it was.

Chen et al. (2018) extended this phenomenon to source memory using a variant of the paradigm in which participants were repeatedly asked to give a congruency judgment based on two temporally spaced (their Experiment 2) color-word features: a color word presented in black font, either followed or preceded by a color patch. Here, both items which are presented on a trial have both a “source” (format: written word or colored square) and a semantic meaning (the color that is represented). Participants completed this congruence task with ease, but when prompted in a surprise trial to choose the color patch they just saw, they could not reliably recall the color that they used to form their judgment (Experiment 2), nor correctly attribute a probe color to its feature source (Experiment 3). Not only were participants unlikely to choose the correct color patch, but they were just as likely to choose the color patch consistent with the color word they had seen. This confirms some intact memory of the recent experience, but loss of key contextual information which would allow the source of a feature to be identified. That is to say that they seem to have intact item memory in that they can recall the semantic representations of the two colors that were presented (which was necessary for the pre-surprise trial task), but no source memory containing information about the format in which each item was presented, hence the term “source amnesia.”

Curiously, in Chen et al.’s Experiment 2 (on which the current studies are based), this chance-level performance is only witnessed when the item that is probed for recall during the surprise test was the item that was presented second. Participants are much more successful at choosing the color when the probed color patch was presented first. This could be taken to reflect that source information is simply better maintained for the first-presented item than the second-presented item because it must be represented strongly enough to persist until the second item is processed to achieve the task goal. Alternatively, Chen et al. argued that this could be attributed to a sort of primacy effect bias, wherein, in the absence of knowledge concerning the sources of the two semantic color items which are held in memory, the semantic representation of the first-presented item is chosen for recall more often than the second-presented item.

None of the working memory models described above handle this result elegantly. It is difficult for TBRS to explain this finding, since TBRS stipulates that forgetting occurs because attention is occupied with something else across a period during which the forgotten information temporally decays; in this paradigm, attention may no longer be focused on the forgotten feature, but it is lost almost instantly. A further issue this finding poses for decay-based theories is that more time has passed since the first-presented item was encoded, yet this item seemingly remains accessible, or is perhaps prioritized. The Embedded Processes model also outlines that information to which attention is paid should be easily accessible for a short time, before time-based decay can act upon it. Therefore, even if the color is not the most highly activated feature when it is probed, because it has been attended so recently, it should be accessible from activated long-term memory. Possibly, making the congruence judgment and/or interpreting the surprise question degrades the representation of the color, either through time-based decay due to the delay, or through interference of new information, but this again does not account for why the most recently presented item is lost while the first-presented item is preserved (also, see the work by O’Donnell and Wyble (2023) supporting the idea that attribute amnesia is not solely caused by interference from a surprise question).

The Resource-Depletion theory seems to partially account for the findings of this paradigm, given its strength in explaining the commonly observed primacy effect. However, a limitation on how much can be encoded (Popov & Reder, 2020) does not seem relevant in this paradigm, because so little information is presented for evaluation in the first place: we expect that this model would predict a working memory capacity much greater than one item (as in Popov, 2023; Popov et al., 2022). Similarly, because the Multicomponent model allows for verbal and visual features to be stored in separate buffers, which would be capable of representing at least one feature at a time, it would not obviously predict that this source information would be lost so quickly and with no competition from more recently presented items. On the subject of competition, the Interference model also seems like it would struggle to explain this loss, as the two “contexts” (here we call them “sources” or “formats”) of written word and color patch seem sufficiently distinct to not be cross-activated and cause interference.

Models allowing for removal of information from working memory (e.g., Lewis-Peacock et al., 2018; Oberauer, 2021) may handle these findings marginally more successfully because they include a mechanism, removal, that not only emphasizes the most relevant information in mind but eliminates the no-longer-needed information. Applied here, because the second-presented feature becomes irrelevant for the expected test as soon as a congruency judgment is reached, the detail could be removed from working memory and forgotten. However, under this logic it remains unclear why participants selectively retain the information which was presented first, as the first-presented feature becomes just as irrelevant to the goal.

Given the major challenge that Chen et al.’s (2018) findings pose for working memory, this phenomenon is important to replicate and to understand more fully before theorists consider whether to adapt their models in response. A gap in the Chen et al. (2018) studies is that they did not test participants’ memory for the verbal information contributing to the congruency judgments. Such an experiment could speak to the generalizability of the effect, which will be important for theorists to take into consideration. Additionally, the results in all of their studies were consistent with the conclusion that source amnesia may not mean that the color is not represented: consistently, observed errors were misattributions in which participants’ choice was consistent with the *word* stimulus that was presented on that trial. These misattributions could indicate, as Chen et al. suggested, that the first-presented feature is more strongly biased for recall, but these findings could also reflect that the verbal feature is more strongly activated, and thus more likely to be selected in surprise tests when the other feature is forgotten.

With the high prevalence of misattributions, which are instances of to-be-ignored information encroaching on target information, it may be useful to draw more explicit parallels between Chen et al.’s paradigm and Stroop interference. Classic Stroop interference occurs when participants struggle to inhibit particularly salient and automatic word-reading tendencies during a color-naming task. In Stroop’s (1935) original study, Stroop interference only occurred naturally in this one direction: words interfered with responses to ink color, but not the reverse. Stroop found that participants required considerable training to develop their color-naming skills and inhibition of word-reading impulses to a sufficient extent to be able to elicit a “reverse Stroop effect” wherein performance in a word reading task was impaired by incongruent text color. This asymmetry of interference is not seen in all variations of the Stroop task: verbal-spatial Stroop tasks, for instance, elicit both the regular (verbal interference on spatial processing) and the reverse (spatial interference on verbal processing) Stroop effect without extensive training (e.g., Virzi & Egeth, 1985), seemingly belying a different relationship between these types of information than between color and word information. It seems that when it comes to interference, a color-word pairing creates quite a unique disparity. This difference in vulnerability to interference suggests that the read word might be more highly activated than the color patch. Drawing a parallel between these two tasks, we suggest that it is possible that the read word would be less susceptible to loss in Chen et al.’s paradigm, whether it is in the first or second position. If greater source amnesia is observed in recall of color information than word information, it would be necessary for models seeking to explain this rapid forgetting to additionally distinguish between the persistence of verbal and visual features somehow.

The working memory models reviewed earlier do not account for the rapid forgetting observed by Chen et al., so it is understandable that they do not necessarily offer explicit insight into what would happen if word, rather than color, were probed in a surprise test. However, using the general assumptions made by each model, we can make suggestions about what potential findings would align with each model. For instance, because the Multicomponent model explicitly distinguishes between verbal and visuospatial storage, we reason that it could predict differential source-related forgetting for visual versus verbal information, due to the different mechanisms and capacities of the different slave systems involved in rehearsing and maintaining information of different types. If word information is not forgotten but color information is, then the Multicomponent model might account for that by expanding on its presumed differences in the durability of representation in these separate, domain-specific stores. Similarly, the TBRS model specifically includes a uniquely verbal memory mechanism, in addition to the domain-general one. Therefore, we expect that TBRS could account for better recall of verbal than for visual source information by appealing to domain-specific resources that are uniquely available for verbal materials. Contrastingly, the Embedded Processes model, the Resource-Depletion theory and Oberauer and Lin’s framework are domain-general in nature, and thus they should not predict a discrepancy between observed source amnesia for verbal or visual information, because the mechanism by which forgetting occurs does not act differently depending on information type. However, it remains the case that, if we observe rapid forgetting of either feature as Chen et al. (2018) observed with color, all models should consider how to explicitly account for those findings.

**Fig. 1:**
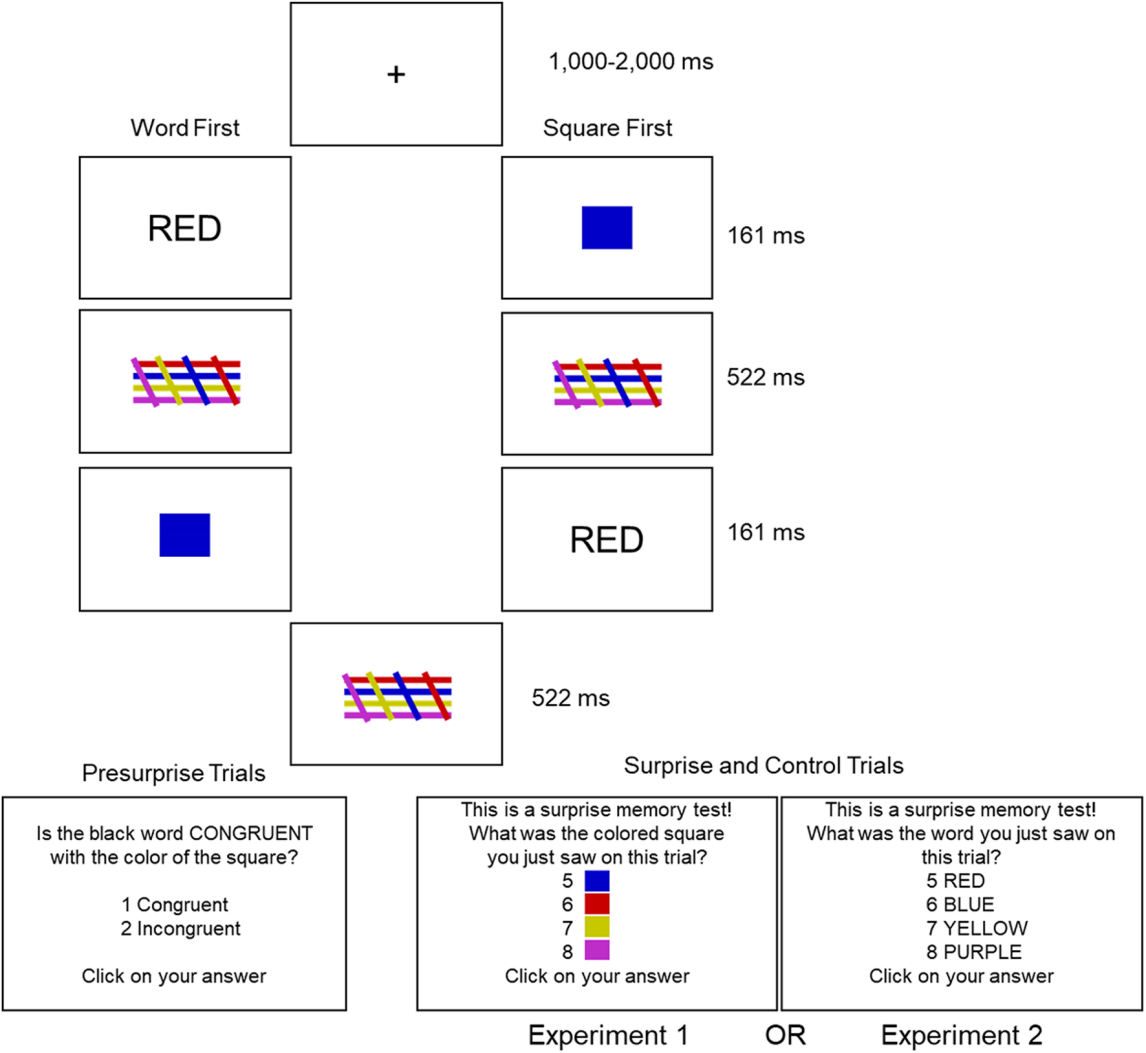
Illustration of the procedure in Experiment 1 and Experiment 2

Here, we address this gap in our knowledge with three experiments: in Experiment 1, we replicated Chen et al.’s Experiment 2 to establish that our method was in line with theirs; in our Experiment 2, we extended the method to test memory for the verbal stimuli; and finally, in Experiment 3, we explored the idea that participants might be encoding a different kind of source than has previously been tested for. Briefly, this method consists of several pre-surprise trials requiring the participant to make a judgment on whether the presented color patch and color word are congruent or incongruent (see Fig. 1 for an illustration). These are followed by a surprise trial wherein the participant is instead asked to report the identity of the color patch (our Experiment 1), the word (our Experiment 2), or the first and second items (our Experiment 3) which they were just shown. In line with previous findings, in Experiment 1 below, we expect to find above chance surprise trial performance when the color patch, which is probed, was presented first in the trial, but chance-level performance when it was presented second in the trial.

## Experiment 1

### Method

In Experiment 1, after many pre-surprise trials consisting of color-word-color-patch congruency judgments, participants were expecting to take part in another congruency test, but instead received an unexpected color memory test. In Chen et al.’s study, participants responded with number keys mapped to response options, whereas in our experiment, participants responded with the mouse by clicking on their chosen answer (both in the congruency judgment pre-surprise trials and in the surprise trial). This adjustment was made in response to the notion that it may be more straightforward for participants.

#### Sample size

We selected a sample size of 20 participants for each condition or group in all experiments, aligning with the sample size used by Chen et al. (2018). This decision was made to ensure reliable estimates across our experiments and to guarantee at least an equivalent number of observations compared to those reported in previous experiments.

#### Participants

In all our experiments, our participants were volunteers recruited via the online data collection agency, Prolific (https://www.prolific.co/). Recruiting via Prolific has been shown to produce comparable data quality in terms of engagement to recruiting university students (whether they take part online or in a lab; Uittenhove, et al., 2023). To participate in our study participants had to meet the following eligibility criteria: (1) native speaker of English, (2) British, American, or Canadian nationality and country of birth, (3) normal or corrected-to-normal vision, (4) no cognitive impairment or dementia, (5) normal color vision, (6) no language-related disorders, (6) aged between 18 and 30 years at the time of sign-up, and (7) with an approval rating of at least 90% on prior submissions at Prolific. All participants were paid £9 per hour (prorated) for their participation in all experiments, which was approved by Cardiff University’s School of Psychology Ethics Committee.

One participant was excluded from analysis due to attaining a pre-surprise trial accuracy of less than 60%. The average age of the participants was 26.5 years (*SD* = 3.01, range 20–31); 46 self-identified as female, 29 as male, three responded that their gender was best represented by the category “other,” and one preferred not to specify their gender.

#### Materials

All experiments were conducted using the online programming software PsyToolkit (Stoet, 2010, 2017). The stimulus design was based on Chen et al.’s Experiment 2 (2018). The verbal stimuli consisted of four different color words displayed in uppercase letters: RED, BLUE, YELLOW, and PURPLE. Verbal stimuli were presented in black, uppercase, 30-pt Arial font at the center of the computer screen on a gray background (RGB values: 150, 150, 150), unless otherwise specified.

Participants were also presented with colored squares measuring 50 pixels by 50 pixels, each displayed in one of four colors: red (RGB values: 200, 0, 0), blue (RGB values: 0, 0, 200), yellow (RGB values: 200, 200, 0), and purple (RGB values: 190, 45, 200). The colored mask was an arrangement of four horizontal lines in each of the four colors, intersected by four diagonal color lines of each of the four colors. The materials and the program are available via the Open Science Framework page associated with this article (https://osf.io/mkwb2/) and the materials described here can be seen illustrated in Fig. 1.

#### Design

The independent variables were as follows: Surprise Trial Congruence (Congruent or Incongruent) and First Stimulus (Word-First or Square-First). The dependent variable was accuracy of color recall, measured using a mouse click. There were four groups of 20 participants. Each group was randomly allocated to one of the four conditions: word-first congruent surprise test, word-first incongruent surprise test, square-first congruent surprise test, square-first incongruent surprise test (see Fig. 1).

#### Procedure

Each participant took part in a single online experimental session lasting approximately 5 min. The procedure (see Fig. 1) was based on Chen et al.’s Experiment 2 (2018) with the following modifications. Each trial began with a variable fixation cross lasting between 1,000 ms and 2,000 ms, immediately followed by the presentation of either the word or the color square (depending on the assigned group) for 161 ms. Subsequently, a mask was presented for 522 ms, followed by the second stimulus (word or color square) for 161 ms. Another mask was then displayed for 522 ms before the test phase.

Before the experiment, participants completed two congruency trials (one congruent, one incongruent) as practice trials in which they received feedback, either “The answer was: Congruent” or “The answer was: Incongruent”. Feedback was not given during the following pre-surprise trials to ensure consistency with Chen et al.'s (2018) methodology. Participants then completed 48 pre-surprise trials of the same structure (24 congruent trials, 24 incongruent trials) with an equal number of trials per color arrangements presented in a random order for each participant. In the pre-surprise trials, participants completed a congruency test, wherein they had to click with their mouse to indicate whether the meaning of the color word presented in black matched the color of the square they saw by clicking on either "congruent" or "incongruent."

These were followed by one surprise trial which was manipulated to be congruent or incongruent, followed by a further four control trials which were randomly selected to be congruent or incongruent. For the surprise and control trials, participants were presented with the following message during the color test: “This is a surprise memory test! What was the colored square you just saw on this trial?” This was followed by the congruency test as they had experienced previously. The order in which the colored squares were displayed during the test phase was randomized. For all tests (congruency and color), participants had up to 1 min to make their decision. After completing all the trials, participants were asked if they had anticipated the surprise memory test: “Were you expecting the surprise memory test where we inquire about the colored square you recently viewed?”, to which they again responded with the mouse by clicking “Yes” or “No.”

### Results and discussion

In the pre-surprise trials, participants took a mean average of 770.881 ms (SD = 1,910.536 ms) to respond across all trials. Participants tended to be very accurate in the pre-surprise with a mean score of 45.911 (SD = 6.611) out of a maximum total of 48, meaning that the error rate was 4.352%. Participants took understandably longer to respond to the color surprise trials, which required new instructions to be read and processed. Here, they had a mean average response time of 5,099.987 ms (SD = 3291.218 ms). In the control trials following the surprise trial, wherein participants likely knew that they would need to recall the identity of the colored square, their error rate was 6.013%.

The key comparison for these data is between the incongruent surprise trial error rates and chance performance. These were calculated by dividing the number of participants who made errors by the total number of participants who took part in each surprise trial type. Chen et al. report 60% and 15% error in their word-first and square-first groups, respectively. In this experiment, our data very closely replicate the findings of Chen et al.’s Experiment 2, with an identical error rate in the word-first and a very similar rate in the square-first trials.

#### Inferential analysis

To compare these results to chance, a chi-squared goodness-of-fit test was conducted, which found that the Incongruent Square-First results did differ significantly from chance (χ^2^(1) = 42.123, *p* < 0.001), but the Word-First results did not significantly differ from chance (χ^2^(1) = 2.400, *p* > 0.05). These inferential results suggest that when the probed item was presented first, its source was remembered, whereas when the probed item was presented second, source information was lost. We decided it would be useful to run these analyses again using participants’ performance on the first control trial as the expected data spread to give a more complete picture of the surprise performance, as was done by Chen et al. (2018). A chi-squared goodness-of-fit test was conducted, which found that the Incongruent Square-First results did not differ significantly from performance on the first control trial (χ^2^(1) = 1.056, *p* > 0.05), but the Word-First results did significantly differ from the first control trial (χ^2^(1) = 127.368, *p* < 0.001). These findings replicate those by Chen et al. (2018) and support that a mouse response is suitable for probing this phenomenon. See Table 1 below for a comparison.

**Table 1:** A comparison of the error data from Chen et al.’s Experiment 2 and the current study’s error data

Error rates	Chen et al		Current study	
	Congruent	Incongruent	Congruent	Incongruent
Word-First	N/A	60%	5%	60%
Square-First	N/A	15%	15%	10.5%

To address the question of misattributions, the number of errors in which the incorrect answer given matched the untested information type for that trial was divided by the total number of errors. Since misattributions were only possible in Incongruent surprise trials, these are the only trials for which data is shown. Our results replicate Chen et al.’s (2018) finding that most errors in the word-first trials were misattributions, but this was the case in much fewer of the errors in the square-first trials. See Table 2 below for a comparison.

**Table 2:** A comparison of the misattribution data from Chen et al. (2018)’s Experiment 2 and the current study’s misattribution data

Trial type	Chen et al		Current study	
	Errors	Misattributions	Errors	Misattributions
Word-First	60%	40%	60%	50%
Square-First	15%	15%	10.5%	0%

These results firmly support the finding from Chen et al. (2018) that source amnesia occurs to a much greater extent when the to-be-recalled item is presented second in a given trial. Our data additionally support their conclusion that misattribution errors attributed to source amnesia are common in this paradigm. This successful replication of previous findings speaks to the robustness of the phenomenon.

## Experiment 2

Having established that the source amnesia results for color memory can be replicated, we used the surprise trial in Experiment 2 to instead test participants’ memory for word information. In Experiment 1, we asked participants only about the identity of the colored square, so the methodology used so far does not allow us to draw firm conclusions about whether the same pattern of forgetting and misattribution would be observed if memory for words was instead tested. The results in all of Chen et al.’s (2018) studies lead to the conclusion that misattribution is a major contribution to the poor performance thought to demonstrate source amnesia. In a control version of their Experiment 2, Chen et al. (2018) removed the response option that corresponded with the unprobed information on the surprise trial and found that participants’ inaccuracy was greatly reduced (down to 10%). Our results from Experiment 1 support this idea, with a huge proportion of the errors made in the Word-First condition, where source amnesia is most common, being misattributions. Misattributions suggest that participants strongly remember the word and are sometimes biased to report it, but do they remember the word information so strongly to the point of commonly misattributing it only because it was presented first, or might they remember the word as strongly regardless of presentation order?

Briefly, as shown in Fig. 1, the only difference in Experiment 2 compared to Experiment 1 was during the surprise trial, wherein participants recalled the identity of the word they were shown instead of the identity of the colored square. Replication of this result in a second domain would speak to the generalizability of the rapid forgetting phenomenon and strengthen the argument for theorists to address this it. We expected that word information might be better recalled than color information, given its special status in the Stroop paradigm, and the unique verbal memory mechanisms which are assumed in some working memory models. If memory for words is more persistent than memory for colors, potential explanations involving domain-specific mechanisms might gain support. However, if word information proves to be no better recalled than color patch information during the surprise trial, we would favor modifying domain-general accounts of working memory to account for rapid forgetting.

### Method

Our Experiment 2 was identical to our Experiment 1 except for the surprise memory test in which we tested recall of the verbal (word) information instead of colors. This manipulation allowed us to investigate whether the pattern established by Chen et al. (2018) and confirmed in Experiment 1 also generalized to verbal information.

#### Participants

In Experiment 2, another group of participants who met the same eligibility criteria described in Experiment 1 and who had not taken part in the previous experiment were recruited from Prolific. Participants were assigned randomly to one of four conditions. Four participants (one in each condition) were excluded on the grounds of not meeting the 60% pre-surprise trial accuracy quota. The final sample was composed of 78 participants. The average age of the participants was 24.8 years (*SD* = 3.12, range 19–30); 29 self-identified as female, 48 as male, and one preferred not to specify their gender.

#### Materials, design, and procedure

The materials, design and procedure in Experiment 2 were identical to Experiment 1 except for the following changes. In Experiment 2, as shown in Fig. 1, the surprise memory test was on verbal information. More exactly, participants were asked to click on which of the four words presented at test was the same as the word that they just saw on that trial (RED, BLUE, YELLOW, PURPLE). The final question of the experiment was also adapted to reflect that procedural change: “Were you anticipating the surprise memory test where we inquire about the word you recently viewed?”.

### Results and discussion

After exclusions based on poor pre-surprise trial accuracy, the mean average score in the pre-surprise trials across conditions was 46.231 (SD = 2.608) out of a total of 48 trials, meaning that the error rate was 3.685%. The mean average response time for these pre-surprise trials was 746.046 ms (SD = 1784.414 ms). Understandably, given the need to read and process new instructions, the surprise trial response time average of 5,554.962 ms (SD = 4282.337 ms) was higher. In the control trials following the surprise trial, wherein participants likely knew that they would need to recall the identity of the color word, their error rate was 8.654%.

Again, the key comparison for these data is between the incongruent surprise trial error rates and chance. These were calculated by dividing the number of participants who made errors by the total number of participants who took part in each surprise trial type.

#### Inferential analysis

To compare these results to chance, a chi-squared goodness-of-fit test was conducted, which found that the Incongruent Word-First results did differ significantly from chance (χ^2^(1) = 29.491, *p* < 0.001), but the Square-First results did not significantly differ from chance (χ^2^(1) = 1.067, *p* > 0.05). When the word information which was probed for recognition was presented first, participants appeared to remember it. However, when this information was presented second, participants performed no better than they would if they were to guess. Again, we ran a second chi-squared analysis on these data comparing participants’ surprise trial performance to their performance in the first control trial. This analysis revealed that the Incongruent Word-First results did not differ significantly from the first control trial performance (χ^2^(1) = 0, *p* > 0.05), but the Incongruent Square-First results did significantly differ from the first control trial (χ^2^(1) = 67.222, *p* < 0.001). See Table 3 below.

**Table 3:** A comparison of the error data for both congruent and incongruent surprise trials when participants were asked to recall the word that they saw (Experiment 2)

	Error rates	
	Congruent	Incongruent
Word-First	10.520%	21.053%
Square-First	10%	65%

In this version of the experiment, we predicted that error rates, and therefore evidence of source amnesia, would be lower than in Experiment 1, due to the comparatively reduced capacity to induce Stroop interference that color information has compared to word information. This prediction is not supported by the results here, with a chi-squared goodness-of-fit analysis suggesting that the error rates did not significantly differ across the two studies for the tested-item-first (χ^2^(1) = 2.235, *p* > 0.05) nor the tested-item-second (χ^2^(1) = 0.875, *p* > 0.05) condition. These results support the idea that this phenomenon is domain general: there is seemingly no difference in the extent of source amnesia when participants are tested on their ability to recall color patches or color words.

The existence of source amnesia that occurs so rapidly poses problems for most models of working memory, but the current results of the phenomenon occurring equally in a second domain lend stronger support to the domain-general models such as the Embedded Processes (Cowan, 1999), Resource-Depletion (Popov & Reder, 2020), and Interference models (Oberauer & Lin, 2017, 2023). Meanwhile, models that suggest that visual and verbal information are stored or maintained differently to each other may find this result more challenging.

Regarding misattributions, the number of errors in which the incorrect answer given matched the untested information type for that trial was divided by the total number of errors. Since misattributions were only possible in Incongruent surprise trials, these are the only trials for which data is shown. From these results, we can conclude that misattributions appear to be roughly as prevalent in word recall as there are in color recall, especially when errors are common. See Table 4 below.

**Table 4:** A comparison of the misattribution data from word-first and square-first incongruent trials when participants were asked to recall the word that they saw (Experiment 2)

Trial type	Errors	Misattributions
Word-First	21.053%	10.526%
Square-First	65%	50%

The misattributions seen in this paradigm may look on the surface to be comparable to the well-documented phenomenon of Stroop interference. First, the stimuli are color words and color squares which are very commonly-used Stroop paradigm stimuli; and second, when participants are asked to recall the color square, we sometimes see a bias towards instead recalling the content of the written word, which mirrors the Stroop effect of failure to inhibit word meaning when responding to visual color information. On the basis that participants struggle much more to inhibit interfering word stimuli during color naming than they do interfering color stimuli during word reading (Stroop, 1935), we hypothesized that misattribution errors might be less common in this paradigm when participants were asked to recall word information than when they were asked to recall color information. The results of Experiment 2 refute this idea, with the rates of errors and witnessed primacy effect being stable across both information types, leading us to conclude that it is unlikely that source amnesia occurs as a result of the same interference documented in Stroop effect research. It seems not to matter therefore which stimulus is causing interference toward the other. Instead, this finding supports Chen et al.’s contention that in this phenomenon, presentation order predicts which feature is dominant in memory: it is the first-encoded feature, regardless of its form. It is possible that these observed error rates will persist in any stimulus type which might be tested, though of course, further study would be required to say this with certainty.

## Experiment 3

Following results from their Experiments 1 and 2 which could equally suggest failure to encode stimulus format as well as they suggest forgetting of stimulus format, Chen et al. (2018) conducted a third experiment. In the surprise trial of this experiment, they showed participants a written word probe, the meaning of which aligned with the colored square which was presented first on that trial and asked them directly to choose whether the color represented by that word was presented in word or colored square format (thus the correct answer was always the “colored square” option). They found that participants were very poor at this explicit version of the task, performing very close to the fifty-fifty level expected by chance despite being always probed on the first-presented item, to which they responded accurately in the previous experiment. From this finding, Chen et al. thus concluded that in this paradigm: (1) The format in which the stimulus is presented is never encoded when it is not known to be needed; and (2) participants are merely biased towards choosing the response which matches the semantic representation of the first-presented item. They suggest that this primacy bias is what leads them to do well in Experiment 2 when the item probed was presented first, and badly when the item probed was presented second.

If Chen et al.’s explanation is correct, and in this paradigm, participants are indeed entirely failing to encode an item’s source when it is not required for the task (though see Wyble et al., 2019, for a discussion on when this is not the case), this would be problematic for the Interference model (Oberauer & Lin, 2017, 2023), which emphasizes that context is the necessary cue which allows items to be recalled. We argue that it is safe to assume that participants can indeed recall the two items presented to them in the surprise trial, given the high prevalence of correct or misattribution answers observed. However, there is a possible alternative that we can see which might allow both Chen et al. and Oberauer and Lin’s suggestions to co-exist in harmony. It is possible that stimulus format is never encoded, but that a different type of context cue *is* encoded. When that context cue cannot be used to answer the surprise question, the primacy bias comes into effect. Commonly suggested types of “context” are an item’s location and an item’s position in serial presentation order. Since all stimuli in this experiment are presented in the same location at the centre of the screen, it is unlikely that location context cues can be effectively used to distinguish them. On the other hand, the stimuli all necessarily have different positions in the serial order. This therefore could be the context cue by which participants are able to access item information in-line with the Interference model.

To test the suggestion that the context through which participants can recall item information in this paradigm is their serial order or position information, another variation of the previously used paradigm was created, with the pre-surprise trials remaining the same, but some key alterations made to the surprise and control trials. During the surprise and control trials, participants were asked which item was presented first and which item was presented second. According to Oberauer and Lin’s model, if serial order is the context cue by which items in the source amnesia paradigm are encoded and retrieved, participants will respond correctly or will extrapolate semantically (choose the response option which aligns with the semantic color representation of the correct response, but in the other format) more frequently than they will misattribute (choose a response in either format which depicts the semantic color which they saw in the not-probed position) or be entirely wrong (guessing) because they have access to correct serial order information. A finding of chance-level performance in this task would be compromising for fundamental assumptions of the model, whereas evidence that participants succeeded in this task would provide very positive support making the Interference model the best contender among the working memory models considered here to explain the source amnesia phenomenon.

### Method

Our Experiment 3 was identical to our Experiments 1 and 2 except for the surprise memory test. In this experiment’s surprise memory test, we asked participants to recall which item was presented first and also which was presented second (order randomized) on that trial. Participants did this by clicking with their mouse on what they believed to be the correct color word or color square item (a total of eight response options instead of four as had been presented in previous experiments). This manipulation allowed us to investigate whether participants had access to a different kind of “source” information than has been tested previously in this paradigm.

#### Participants

In Experiment 3, another group of participants who met the same eligibility criteria described in Experiments 1 and 2, and who had not taken part in the previous two experiments were recruited from Prolific. Participants were assigned randomly to one of two conditions (their surprise trial was either square-first or word-first). For each condition, the presentation of the test order was counterbalanced across participants to control for order effects, but these were collapsed to form two groups of 80 participants each. Consequently, the sample was larger than in previous experiments. One participant (from the square-first condition) was excluded on the grounds of not meeting the 60% pre-surprise trial accuracy quota. The final sample was composed of 159 participants. The average age of the participants was 26 years (*SD* = 3.72, range 19–30); 92 self-identified as female, 61 as male, five as a different gender, and one preferred not to specify their gender.

#### Materials, design, and procedure

**Fig. 2:**
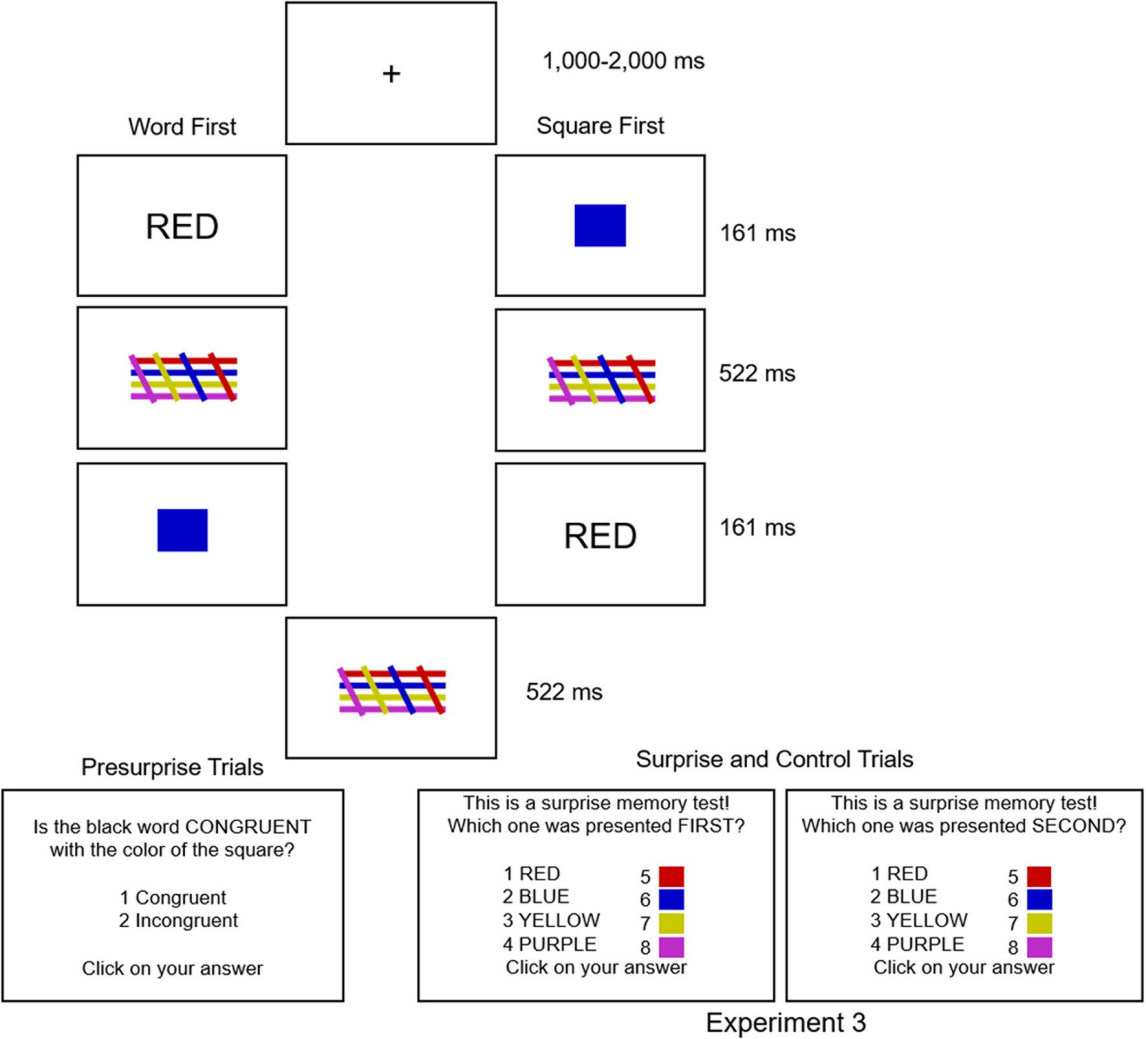
Illustration of the procedure in Experiment 3

The materials, design and procedure in Experiment 3 were identical to Experiments 1 and 2 except for the following changes. In Experiment 3, as shown in Fig. 2, the surprise memory test asked participants to recall the first- and second-presented items (order counterbalanced). More exactly, participants were asked to click on which of the four words and four colored squares presented at test were the same as the first and second items that they just saw on that trial (RED, BLUE, YELLOW, PURPLE). The final question of the experiment was also adapted to reflect that procedural change: “Were you anticipating the surprise memory test where we inquire about the which one was presented first or second?”.

### Results and discussion

After exclusions based on poor pre-surprise trial accuracy, the mean average score in the pre-surprise trials across conditions was 46.396 (SD = 2.670) out of a total of 48 trials, meaning that the error rate was 3.342%. The mean average response time for these pre-surprise trials was 708.500 ms (SD = 1,480.758 ms). Understandably, given the need to read and process new instructions, the surprise trial response time average of 7,112.607 ms (SD = 6988.999 ms) was higher. In the control trials following the surprise trial, wherein participants likely knew that they would need to recall the positions of the stimuli, the percentage of participants selecting either the precisely correct or “semantically correct” answers (answers which had the same meaning as the precisely correct answer, but in the incorrect stimulus format) was 65.566% across all conditions and both positions. This demonstrates that participants could complete the task if they knew that they would be asked to do so.

#### Inferential analysis

Again, chi-squared analyses were conducted to compare the observed spreads of data for each condition to the spread which would be predicted by chance responding. The observed distribution of frequencies in the square-first group did not significantly differ from chance (for which the expected response proportions would be 12.5%, 12.5%, 25%, and 50%, in line with the order of response types in Table 5) regardless of whether they were tested on the identity of the first-presented item, the square, (χ^2^(3) = 3.860, *p* > 0.05), or the second-presented item, the word, (χ^2^(3) = 5.250, *p* > 0.05). It is the same situation in the word-first group: neither the results for the first-presented item, the word (χ^2^(3) = 4.150, *p* > 0.05), nor the second-presented item, the square (χ^2^(3) = 4.250, *p* > 0.05) differed significantly from chance. Additionally, we ran chi-squared analyses to compare surprise trial performance to performance on the first control trial. For the square-first group, these distributions differed for both the square (χ^2^(3) = 71.130, *p* < 0.001) and the word (χ^2^(3) = 50.037, *p* < 0.001). This pattern was the same for the word-first group for both the word (χ^2^(3) = 120.864, *p* < 0.001) and the square (χ^2^(3) = 107.345, *p* < 0.001).

**Table 5:** A comparison of the proportions of responses for both word-first and square-first surprise trials when participants were asked to recall the first and second items that they saw (Experiment 3). “Correct” refers to responses which selected the same semantic meaning and stimulus format as was presented on that trial. “Semantically correct” refers to answers which had the same meaning as the precisely correct answer, but in the incorrect stimulus format (e.g., if correct response would be the blue square, the word BLUE was chosen instead). “Misattribution” refers to answers which corresponded to the non-probed item presented on that trial, regardless of stimulus format. “Guess” refers to answers which did not correspond with a stimulus presented on that trial, belying random guessing

	Percentage of response types							
	Testing word				Testing square			
	Correct	Semantically correct	Misattribution	Guess	Correct	Semantically correct	Misattribution	Guess
Word-First	12.500%	13.750%	33.750%	40%	17.500%	6.250%	23.750%	52.500%
Square-First	7.595%	16.456%	32.911%	43.038%	13.924%	16.456%	30.380%	39.241%

Chance performance primarily indicates that participants did not know which stimulus was presented in which position during the surprise trial, refuting the hypothesis that serial order position information is being encoded in this paradigm. Even when expanding our definition of “correct” answers and taking semantic extrapolation responses into account (where participants knew which color semantically was presented but selected the wrong format, e.g., *blue square* when the answer was “blue”), participants’ performance is not indicative that they could use the correct serial order position cues to recall the items they saw. These results taken with the previous experiments reported here support Chen et al.’s (2018) notion that no feasible type of context or “source” is encoded in this phenomenon. This is problematic for the Interference model (Oberauer & Lin, 2017, 2023) as discussed earlier, because without a linked context, the model predicts that items should not be accessible in working memory, but in some select instances (e.g., when the first item is probed by format in Experiments 1 and 2), the information is accessible.

A counter to this argument might be made in the form of the Interference model’s Focus of Attention element, which is proposed to confuse the context-content links of items held within it at the same time (Oberauer & Lin, 2017). If the stimuli in this paradigm are thought to be held in the focus of attention simultaneously, their links would be confused regardless of which context type they consisted of, and they would not be expected to know which item was presented in which format (Experiments 1 and 2), nor in which position (Experiment 3). These findings therefore argue strongly for the inclusion of the focus of attention element in this model for maximum explanatory power. This is an important argument because the inclusion of this element of the model has previously been debated following mixed results from testing model fits (Oberauer & Lin, 2023). Alternatively, perhaps this finding warrants a clearer definition of what can and cannot be considered a “context” in the model. For instance, could the stimuli in this paradigm be linked to the planned congruent/ incongruent response which participants intend to make about them?

It is additionally interesting that the results of this final experiment suggest a total loss of item information: participants select response options that correspond with one or the other of the presented stimuli just as often as they would if they were guessing. This was not predicted by either the Interference model (even with a focus of attention adjustment), nor the primacy bias suggestion made by Chen et al. (2018). If the stimuli are proposed to be held in the Interference model’s focus of attention, they should be more or less guaranteed to be accessible on a semantic level. If participants are biased to report the first-presented item, regardless of source, why do they guess randomly in this instance? Further, this finding is in stark contrast to the previous results reported here wherein the prevalence of incorrect, non-misattribution responses (i.e., guesses) has consistently been in the realm of 10–15%, much lower than the 50% guess rate expected by chance in those previous experiments (where two of four possible response options were correct or misattributions).

It is possible that this inconsistent result is due to the introduction of extra response options. In this third experiment, participants chose between eight instead of four response options, which could feasibly be overwhelming and either delay responses to the point where time-based decay might have the chance to act (the average surprise trial response time was higher in this experiment than in the previous two by about 1,500 ms), or cause interference as extra items which must be processed before the response can be made. Or it could be simply that participants were asked explicitly about the order in which items were presented, and this cued their recall very poorly when they expected to judge congruency. Whatever the mechanism, clearly this change had a strong negative effect on participants’ performance compared to previous experiments, to the point where they could no longer reliably recall which two semantic items they saw. An important takeaway from this study is that we may still not fully understand the impact of surprise questions on memory performance or the factors which mediate this effect.

## General discussion

To review, the three studies reported here had two major aims: First, to replicate and extend previous research to investigate whether the extent of source amnesia would differ depending on the type of information which was tested. This subject is of high theoretical interest because replication of such a phenomenon in a second domain is very convincing of its importance for accommodation in memory models. Alternatively, if the finding had replicated in Experiment 1 when memory for color items was tested but not in Experiment 2 when memory for verbal items was tested, this might have spoken to an essential difference between these stimulus types which would also need to be explained by models hoping to accommodate this phenomenon. The finding of a disparity between information types would also have mirrored the well-established Stroop interference disparity with the same information types (Stroop, 1935) and might have indicated similar underlying mechanisms in these two phenomena, opening avenues for better understanding of both. The second aim of this study was to test whether participants would be able to successfully identify which item was presented first and which was presented second, which would indicate that they were encoding the context of position in presentation order instead of stimulus format (colored square or written word). The implications of the findings of the final experiment are important for the Interference model (Oberauer & Lin, 2017, 2023), which emphasizes that associated context information is essential for the recall of an item.

In Experiment 1, our results closely replicated the findings of Chen et al. (2018): that source amnesia occurred to a greater extent when the probed information type was the one which was presented second in the trial and that the majority of errors were misattributions. Our novel finding from Experiment 2 is that source amnesia occurred to a very similar extent when participants were asked to recall the source of word information. Additionally, the proportions of errors which can be labelled as misattributions were very similar across the two experiments. We therefore conclude that regardless of whether color or word memory was tested, participants were likely to choose the option at test which was consistent with the meaning of the first-presented feature. Replicating a phenomenon such as this in a second domain bolsters its credibility and strengthens the argument for models of working memory to be amended to accommodate these findings. In addition, the chance-level results from our Experiment 3, which tested participants’ memory for order information, lead us to conclude that no form of context which we can see is necessarily encoded alongside semantic representations of item memory when presented so rapidly.

Though it is interesting that these semantic representations appear to be very susceptible to loss, with participants guessing at random from the eight response options during Experiment 3, seemingly having lost even the previously preserved semantic item memory of what they had just seen. This particular finding leads us to wish for a better understanding of the factors influencing the impact that surprise questions can have on participants’ memory performance. A study by O’Donnell and Wyble (2023) has already begun to address this and has concluded that while surprise questions do have an impact on participants’ memory performance, this cannot account for the magnitude of information loss in source and attribute amnesia. Further, Muter (1980) compared trigram recall performance following a distraction task when participants were surprised with the recall prompt to when they were made aware from the beginning that this would occur on a small number of trials. They reported no notable difference in performance as a result of knowing that they would experience these “surprise”-esque trials, which implies that the element of surprise is not likely to cause the forgetting they witness in their method, and perhaps by extension, in this paradigm. In light of our novel finding, it seems that there is more to be uncovered on this subject and that it warrants more in-depth further study.

## Addressing the models

Following the experiments detailed here, we are more confident that participants do not encode the source nor any obvious context for the items they observe in this paradigm. For the task that participants intend to carry out (the pre-surprise task), they do not need to know which form the information they process was presented in: they only need to compare the semantic meanings of the stimuli they observe. We believe that the mind often conserves its resources where possible, and since source information is not needed in the pre-surprise task, it stands to reason that instead of being forgotten, it may purposefully not be encoded at all (an idea which has already been explored in Chen & Wyble, 2016). This is a problematic assumption for the Embedded Processes model, which posits that all attended information enters the focus of attention (and therefore activated long-term memory) at least briefly, and thus there should be some trace of source information accessible after so short a period. The Embedded Processes model could adjust to allow for rapid forgetting by introducing new boundary conditions on entry to the focus of attention and/or allowing for de-activation of long-term memory under these circumstances. This is also potentially an issue for the Interference model, which, as discussed earlier, would argue that without associated context information, items should not be retrievable from memory. One would expect that a failure to encode source information would be problematic for the Multicomponent model because it would necessarily assign the verbal item to the verbal short-term store and the visual item to the visual short-term store, meaning that their source would be inherent depending on the store in which they are maintained. The same could be said for TBRS model here, given their suggestion of a verbal-only memory mechanism – if an item is being stored by that mechanism, it follows that it was presented in a verbal format. The Resource-Depletion theory suggests that unless context-item bindings are necessary, cognitive resource is not dedicated to forming them (Popov & Reder, 2020). This seems to be the case in this example, but this claim is discordant with the wealth of literature documenting the occurrence of incidental bindings (e.g., Campo et al., 2010; Elsley & Parmentier, 2015; Logie et al., 2011; Morey, 2011; Santana & Galera, 2014; Treisman & Zhang, 2006), so perhaps there is room to elaborate in this model which circumstances do and do not permit incidental binding when it is not explicitly called for.

Our results from Experiment 2 align with some of the findings by Xu et al. (2020), who used a similar methodology of visually presenting words. Interestingly, however, our findings diverge from theirs in their experiment wherein the words were presented auditorily, as they did not observe rapid forgetting. This suggests that memory for visually presented words is more susceptible to rapid forgetting compared to spoken words. At first glance, these results may seem difficult to reconcile with existing memory models. However, they align well with established phenomena such as the modality effect (Watkins & Watkins, 1977, 1980), the superior memory performance for recently presented items when information is presented auditorily rather than visually. Thus, our findings, along with those of Xu et al. (2020), may be reconciled with memory models that propose auditory presentations have distinctive characteristics that make them more resistant to forgetting or interference at least across periods this brief (e.g., Nairne, 1990; Saint-Aubin et al., 2021). Nevertheless, future research will be needed to directly evaluate this proposition.

An alternative reason as to why context may not be encoded in this paradigm could be that it is a result of the stimulus presentation rate. Popov et al. (2022) found that the binding of some items (low-frequency words) to contexts (locations) was worse at very fast presentation rates (500 ms compared to 750 ms and 1,000 ms). In the current experiments, stimuli were presented for even less time, perhaps suggesting that in some cases, it may be a natural consequence that item-context bindings are not made if presentation times are too brief. Further support for this may come from the Attentional Blink phenomenon frequently observed in experiments of the Rapid Serial Visual Presentation (RSVP) paradigm, which consistently show that at very fast list presentation times (e.g., 107 ms per item), a second target for detection and later recall is often missed if presented between approximately 200–500 ms after the successfully detected first target (Broadbent & Broadbent, 1987; Nieuwenstein & Potter, 2006; Potter et al., 2010). These findings could be taken to indicate that during a specific time window following encoding of the first target, the second target is not successfully bound to the context (which is what gives it its target status among the distractors, e.g., the color of the letter item or being marked by some punctuation indicator). In the RSVP task, the presence of multiple non-target distractors may mean that the item information for the second target is confused with distractors before recall can occur at the end of the list, but in this source amnesia paradigm where there are no distractors (only a brief mask), both items are remembered, and it seems that only the source is forgotten.

An alternative explanation to the failure to encode argument is that once information is removed from our focus, it may be specifically inhibited or suppressed to aid in task switching or conserve cognitive resources. This idea is discussed by Lewis-Peacock et al. (2018). In the current paradigm, if the second-presented item is removed from focus and specifically suppressed in favor of generating and holding a response plan to the pre-surprise trial incongruency judgment task (which is what participants would expect to do in the surprise trial before they see the new instructions), this might explain why memory for that second-presented item is poorly accessible. One could argue that this suppression would equally apply to the first-presented item and that it would be even harder to access given that it was presented earlier, but this might be counter-acted by some level of short-term consolidation (Jolicœur & Dell’Acqua, 1998) which was carried out to hold the first-presented item during the very short mask between first and second items. Our confirmation in Experiment 2 that the preservation of the first-presented item occurs for verbal as well as visual features underscores the need to think further about potential boundary conditions on proposed maintenance processes in working memory. For example, complete removal might be more likely for information that has not yet been encoded to a particular degree, or perhaps has not figured into any plan.

## Addressing primacy bias

Chen et al. put forward the idea of a primacy bias, which is not unsupported by the working memory literature: the primacy effect in memory (Oberauer et al., 2018) is a well-replicated effect which is often targeted for explanation by models. However, here Chen et al. would argue specifically that it is not the source of first-presented items is remembered, but instead that participants are biased to report the semantic representation of the first-presented item more often than that of the second-presented item. This is an incomplete explanation however, as it stands to reason that they should not only be blindly biased to report the first-presented item when the first-presented item was probed: they should “guess” the first-presented item to the same extent whether they are in the word-first or the color-first condition. This is not what is seen in their Experiment 2, however: only in the square-first condition is the first-presented item most likely to be chosen. Additionally, in the Experiment 3 reported here, no such primacy bias was witnessed when the paradigm was altered very minorly to ask participants about serial order positions instead of stimulus format. An explanation is needed which accounts for this asymmetry of response better than an omnipresent bias towards the first-presented item. Perhaps in the source-probing version of the paradigm, some proportion of participants actually know the answer and there is a bias towards the first-presented item only in the case that a participant is unsure.

A particular strength of Popov and Reder’s (2020) Resource-Depletion model is that it tidily explains the primacy effect in serial recall memory with its resource depletion mechanism (although see Popov, 2023, for discussion of a phenomenon within the primacy effect literature which does pose a problem for the model as it stands). The model states that the amount of resource dedicated to encoding each subsequent item declines as less resource is available for the task, and that the less resource that is dedicated to encoding an item, the less easily it is retrieved. This seems to provide a good account for the primacy bias here: with such a short delay between presentation of the first and the second item, there would assumedly be very little opportunity (if any) for resource recovery, and thus we would expect the first item to be better recalled than the second. In addition, it is unclear what possible explanation this model could suggest for the knock-out effect which occurred in our Experiment 3 when participants were asked for serial order information instead of source information. Why would participants not be inclined again to rely on the primacy bias which they had used so consistently in the first two experiments? Surely with such emphasis in this model on the superiority of the first-encoded item, we would expect our participants to do very well when asked for the identity of that item.

We conclude that at very short presentation times, participants do not automatically encode any form of context when they do not require it for the task at hand. The performance data from our control trials and those reported in published literature in this and other realms of extremely rapid forgetting (Chen & Wyble, 2015, 2016; Chen et al., 2018) suggest that participants *can* maintain this context information when they believe that they need to do so. This therefore implies that there is some cost associated with encoding context information during such brief stimulus-presentation time periods. Working memory is ultimately for action in service of some goal. Perhaps, besides attention-based assumptions about what is encoded, models should focus on the fate of information prioritized for responding, emphasizing why that seems to differ from more incidental details.

## Data Availability

All materials, program, data and the analysis scripts for this study are available at the OSF page (https://osf.io/mkwb2/). None of the experiments reported here were pre-registered.

## Code availability

The program code for this study is available at the OSF page (https://osf.io/mkwb2/).

## Declarations

### Open Practices Statement

All materials, program, data and the analysis scripts for this study are available via the Open Science Framework (OSF) page (https://osf.io/mkwb2/). None of the experiments reported here were pre-registered. While working on this manuscript, DG was supported by EPS small grant and NC by NIH Grant R01-HD21338.
